# Risk Factors of Secondary Bacterial Infection in Patients Hospitalized with COVID-19

**DOI:** 10.3390/jcm15114126

**Published:** 2026-05-27

**Authors:** Agnieszka Bednarska, Marek Radkowski, Tomasz Laskus, Anna Furman-Dłubała, Michał Makowiecki, Dawid Porowski, Justyna D. Kowalska, Marcin Paciorek

**Affiliations:** 1 Department of Adults’ Infectious Diseases, Medical University of Warsaw, 01-201 Warsaw, Poland; 2 Hospital for Infectious Diseases in Warsaw, 01-201 Warsaw, Poland; 3 Department of Immunopathology of Infectious and Parasitic Diseases, Medical University of Warsaw, 3C Pawińskiego, 02-106 Warsaw, Poland; 4 Center for Observational Research in Infectious Diseases, Medical University of Warsaw, 01-201 Warsaw, Poland

**Keywords:** secondary, bacterial, COVID-19, risk factors

## Abstract

**Background/Objectives**: Secondary bacterial infections in subjects hospitalized with COVID-19 are associated with longer hospital stays and an increased risk of death. Thus, identifying the risk factors associated with bacterial complications in patients with SARS-CoV-2 infection is clinically important. **Methods**: We analysed the collected records of all adult patients diagnosed with an acute COVID-19 infection who were admitted to the Hospital for Infectious Diseases in Warsaw, Poland, between March 2020 and November 2021. Logistic regression models were used to identify factors associated with bacterial infections complicating the SARS-CoV-2 course. **Results**: The records of 1963 patients were analysed; 1128 (57.4%) of the patients were male, the median age of the cohort was 63 years (IQR: 50–74 years), and 202 patients (10%) died. A bacterial infection complicating the course of COVID-19 was diagnosed in 351 (18%) patients. In a multivariate logistic regression model the only factors identified as independently associated with an increased odds of bacterial infection were age (OR per decade: 1.21, *p* = 0.016), length of hospitalization (OR per day: 1.12, *p* < 0.001), and D-dimer concentration (OR per 500 units: 1.01, *p* = 0.048). **Conclusions**: Shortening the length of hospitalization of patients with COVID-19 can reduce the risk of bacterial infection complications, especially in elderly individuals. Coagulation system activation in COVID-19 patients increases the risk of a bacterial infection.

## 1. Introduction

The SARS-CoV-2 virus primarily infects cells in the respiratory system and, similarly to other viral infections, is associated with an increased risk of a secondary bacterial infection, which has a worse outcome than either infection on its own [1,2]. After adjustment for demographic factors and comorbidities, the risk of death is 2.3 times higher than that in patients with negative bacterial testing [3].

In patients with COVID-19, bacterial infections are associated with a longer hospital stay, and the odds of death were found to be increased up to sixfold [4,5]. In another study, COVID-19 patients with bacterial co-infections were found to have a 25% risk of death at 30 days of hospitalization [6]. Moreover, when COVID-19 is compared with other respiratory tract viral infections, the odds of secondary bacterial infections in patients with COVID-19 are higher [7], with the estimated cumulative risk of developing at least one such episode being 25% to 50% [8]. The respiratory tract is the most common site of secondary bacterial infection, and severe cases of SARS-CoV-2 infection are more susceptible to multidrug-resistant pathogens, leading to increased mortality rates [9].

The results of studies on underlying factors related to bacterial complications are not consistent, most likely due to differences in the factors considered across the studies [10,11,12]; furthermore, the World Health Organization (WHO) reported on the extensive overuse of antibiotics during the COVID-19 pandemic [13].

In the current analysis we aim to analyse the factors that could be associated with the development of bacterial infections in patients hospitalized with COVID-19.

## 2. Materials and Methods

We analysed data collected for a cohort of adult patients (≥18 years old) diagnosed with COVID-19 and admitted to the Hospital for Infectious Diseases in Warsaw between 6 March 2020 and 30 November 2021. The qualification procedure for hospitalization was as follows: Individuals with symptoms suggestive of COVID-19 were consulted at the Emergency Department, and the diagnosis of COVID-19 was confirmed by real-time reverse transcriptase polymerase chain reaction (RT-PCR) and/or SARS-CoV-2 antigen detection from nasopharyngeal swabs. Only patients with a confirmed SARS-CoV-2 infection and who required oxygen therapy were admitted to hospital.

The clinical and epidemiological data were collected as a part of routine care. The hospital adopted Standard Operational Procedures (SOPs) specific for COVID-19 and amended the software to allow for a new dataset collection.

A questionnaire that included information about comorbidities, the results of a physical examination, applied methods, and treatment outcomes, specifically designed for the purpose of this study, was employed to collect clinical data. A standardized set of laboratory tests were performed. The data collected during the medical examination were verified and, after encoding and the removal of sensitive personal data, entered by a physician into a web application containing an electronic form adapted to the collection of data necessary for the implementation of the project. The data were stored in the application database in an anonymized, coded and standardized manner. Laboratory results were imported directly from the hospital information system into the application.

For the purpose of the present study we analysed clinical data and laboratory and radiological test results on admission. Only patients with secondary bacterial infections were enrolled in the study, and the presence of a bacterial infection was diagnosed based on clinical, laboratory, radiological and microbiological testing according to the ECDC case definition [14].

Univariate and multivariate logistic regression models were used to identify variables associated with bacterial infections. Variables were considered significant if the *p*-value for the Wald test was strictly below 0.05. Variables that were missing in ≥20% of patients were not included in the multivariate analysis. All computations were done using R version 4.1.1.

This study was approved by the Bioethics Committee of the Medical University of Warsaw (approval number: AKBE/154/2022).

## 3. Results

### 3.1. General Results

A bacterial infection was diagnosed in 351 (18%) out of 1963 patients. The demographic, clinical and laboratory data are presented in Table 1; 1128 patients (57.4%) were male, and the median age of the cohort was 63 years (IQR: 50–74 years). Overall, 202 out of 1228 patients (10.3%) died; the mortality rate was 20.5% (72 patients out of 351) in the group with secondary bacterial infection and 8.1% (130 patients out of 1612) in the non-bacterial group.

The most common symptom of COVID-19 was cough (80%), followed by weakness (75%), fever (75%) and dyspnoea (62%). The most common comorbidities were hypertension (50%) and diabetes mellitus (19%), and 31% of individuals were obese, with BMI > 30 (Table 1).

The median oxygen saturation without oxygen therapy was 93% (IQR: 88.0–97.0%), and high-resolution computed tomography (HRCT) showed lung injury with a median of 30% (IQR: 20–50%) tissue involvement. The median SOFA score was 1.0 (IQR: 0.0–2.0) in the whole cohort and 2.0 (IQR: 1.0–3.0) in patients with a bacterial infection. During hospitalization, as an adjunctive treatment, 83.5% of the patients received low-molecular-weight heparin, and 63% received steroids. The latter treatment was more common among patients with a secondary bacterial infection (82% vs. 59%). The median length of hospitalization was nine days (IQR: 6.0–13.0 days) for the whole cohort and fifteen days (IQR: 10.0–22.0 days) for patients with a secondary bacterial infection (Table 1).

### 3.2. Univariate Logistic Regression

In the univariate logistic regression model the factors associated with increased odds of acquiring a secondary bacterial infection were age, male gender, working in a health care facility, baseline low oxygen saturation, SOFA score on admission, length of hospitalization and the presence of a fever, myalgia, a sore throat, a runny nose, a cough, loss of taste and smell, or abdominal pain; among the comorbidities it was dementia, COPD, diabetes, paresis, neoplasm, interstitial lung disease, hypertension, atrial fibrillation and immunosuppressive treatment that were associated with bacterial infection. Furthermore, bacterial infection was associated with lung involvement documented by high-resolution computed tomography, the concentration of C-reactive protein, lactate dehydrogenase, urea, D-dimer, interleukin-6, NT-proBNP, the number of neutrophils and EGFR < 60 mL/min/1.73 m^2^. Considering the clinical characteristics, factors such as arrhythmia, cardiovascular and thromboembolic complications, and treatment with steroids and heparin were identified (Table 1).

### 3.3. Multivariate Logistic Regression

In the multivariate logistic regression model (Table 2) the only factors identified as independently associated with increased odds of a bacterial infection were age (OR: 1.21, *p* = 0.016), length of hospitalization (OR: 1.15, *p* < 0.001), and D-dimer concentration (OR: 1.01, *p* = 0.048).

## 4. Discussion

The prevalence of bacterial co-infection and secondary infection in COVID-19 patients ranges from 6 to 16% [5,10,12,15,16], and a severe course of COVID-19 is reported to be a predisposing factor [17,18]. In the current study, which included 1963 hospitalized patients, a bacterial co-infection was diagnosed in 351 (18%). The latter group was characterized by a more severe clinical course. On admission, bacteria-coinfected patients presented with lower oxygen saturation, higher D-dimer and IL-6 concentrations, and more widespread lung tissue involvement.

Among our patients, age was a significant risk factor for bacterial infections, in both univariate and multivariate analyses, increasing the risk by 20% with every ten years. This is not unexpected since, in elderly individuals, innate and adaptive immune systems undergo changes defined as inflammaging and immune senescence, and this leads to increased vulnerability to viral, bacterial, and opportunistic infections [19]. The risk of having a severe infection increases with age, and a high comorbidity score worsens the situation [20]. The patients in this study presented with a higher number of comorbidities, including diabetes and cancer, which are well recognized as immunosuppressive factors enhancing susceptibility to bacterial co-infections [9,12,21,22].

Another independent factor associated with bacterial co-infection in our study was the length of hospital stay (LOS). We estimated that a prolonged length of hospitalization increased the risk of gaining a bacterial infection by 12% every day. The hospital setting is a well-established risk factor for infections due to environmental contamination, the use of invasive devices like central lines and catheters, and the stress of hospitalization itself [23,24,25]. Susceptibility to infections could be further impaired by an immunosuppressive treatment: the patients whose in-hospital stay was complicated by a secondary bacterial infection were more likely to have a history of an immunosuppressive treatment before admission and were more likely to receive steroids as an adjunctive treatment during hospitalization. However, these factors were significant only in a univariate analysis.

Similarly, such comorbidities as dementia, diabetes mellitus, neoplasm, atrial fibrillation, hypertension and paresis were found to be associated with bacterial infection in our study. The association between dementia and secondary bacterial infection was especially high (OR 4.15) and could be influenced by a variety of medical, social, and organizational factors, like cognitive decline leading to poor hygiene, dysphagia and a risk of aspiration, immobility or delayed health-seeking behaviour. Indeed, comorbidities, including dementia, obesity, chronic liver disease, haematological malignancies and venous thromboembolism, have been reported to be predictors of prolonged hospitalization [26]. However, our analysis revealed such a link only in a univariate model. This suggests that it was the prolonged hospitalization and not the co-occurring conditions that were responsible for these infections.

In a previous study it was found that extending the LOS by one day increased the probability of acquiring a nosocomial infection by 1.37 percent [27]. As already mentioned, the probability of bacterial complications in our cohort was higher and estimated at 12% with every day of being hospitalized, which might stem from the specificity of the coronavirus infection. Coronavirus-induced airway damage promotes the attachment and systemic dissemination of bacterial pathogens [28,29]. This was reflected in epidemiological studies with data showing a continued increase in healthcare-associated infections during the COVID-19 pandemic [25].

Another independent factor associated with bacterial infection among our patients was the level of D-dimer in the blood. High D-dimer values in patients with COVID-19 were previously found to be strongly associated with unfavourable clinical outcomes, specifically with higher rates of intubation and mortality [30,31]. COVID-19 patients often exhibit an exaggerated thrombolytic response to the viral infection, but coagulation plays a pivotal role in bacterial infections as well [32,33,34].

The process of immunothrombosis is a major element of the intravascular innate immune system. Clustering pathogens circulating in the blood through the formation of microthrombi prevents their dissemination and tissue invasion. The microthrombi serve as a platform that facilitates the recognition and destruction of pathogens. The microvascular accumulation of fibrinogen and fibrin promotes the recruitment of additional immune cells to the site of a tissue infection, further supporting the immune response [34,35]. Thus, such severe bacterial infections as endocarditis or bloodstream infections can be effectively identified by high levels of D-dimer [36].

Although immunothrombosis is suggested to support pathogen recognition and host defence, when dysregulated, it may lead to severe thrombotic events. In severe COVID-19, excessive inflammation, driven by cytokine release, contributes to intravascular coagulopathy by promoting endothelial dysfunction and the recruitment of neutrophils [37]. Activated neutrophils extrude a web of DNA-based cytoplasmic material containing antimicrobials—neutrophil extracellular traps (NETs). While NETs represent a defensive strategy against invading microbes, when disrupted they may increase vascular permeability and accumulate activated platelets, leading to immune defence dysregulation [38,39] and thus preventing successful bacterial clearance. A higher number of neutrophils increasing the risk of bacterial infection in the univariate analysis reflects this phenomenon. The more common use of dexamethasone—which is known to suppress neutrophil adhesion to endothelial cells, impair lysosomal enzyme release, and inhibit migration to inflamed sites—in the group with a higher risk of secondary bacterial infection might be an additional explanation [40,41]. Male sex was found to be a protective factor against secondary bacterial infection in the univariate analysis. In general, females have stronger innate and adaptive immune responses but also are more prone to inflammatory and autoimmune diseases [42]. Whether this phenomenon reflects dysregulated immunity in female patients, predisposing them to a higher risk of secondary bacterial infections during the course of COVID-19, warrants further investigation.

Finally, anosmia, ageusia, rhinorrhoea, and a sore throat were negatively associated with secondary bacterial infection in our study. An early interferon (IFN) response to SARS-CoV-2 in the upper respiratory tract is associated with the development of a symptomatic infection through increased cytokine and chemokine secretion [43]. On the other hand, IL-13 reduces ACE2 expression, the intracellular viral load, and cell-to-cell transmission [44]. Since IFN reduces protein levels of IL-13 [45], it can be speculated that patients who develop early (upper respiratory) symptoms might better control the infection, prevent its spread, and consequently avoid bacterial complications.

Our study is not without limitations. It was conducted in a specialized hospital and, therefore, patients could be preselected according to a certain severity. The accessibility of hospital care during the COVID-19 pandemic was significantly changing. Due to the severity of COVID-19, bacterial infections were diagnosed based on clinical, laboratory, and radiological testing, not only microbiological testing.

## 5. Conclusions

In conclusion, we analysed a large group of inpatients with COVID-19 and demonstrated that the duration of hospital stay, advanced age and fibrinolytic activity reflected by D-dimer levels were independent predictors of secondary bacterial infection. To reduce the risk of bacterial complications the duration of hospital stay should be kept to a minimum, particularly among older patients. We hope these findings contribute to optimizing patient management and clinical outcomes.

## Figures and Tables

**Table 1:** Baseline characteristics of patients with/without infectious complications, and univariate logistic regression model of factors related to developing infectious complications during hospitalization.

Univariate Analysis OR (OR CI), *p*-Value	Bacterial Infection n = 351	No Bacterial Infection n = 1612	All n = 1963	Characteristics
Demographic data				
1.52 (1.40–1.65), *p* < 0.001	7.3 (6.1–8.3)	6.2 (4.8–7.2)	6.3 (5.0–7.4)	Age (decade), median (IQR)
0.75, (0.60–0.95), *p* = 0.017	182 (51.7)	946 (58.6)	1128 (57.4)	Male gender, n (%)
1.0 (0.98–1.01), *p* = 0.86	7.0 (5.0–10.0)	8.0 (6.0–11.0)	8.0 (6.0–11.0)	Number of days from the onset of the first symptoms to admission, median (IQR)
1.15 (1.13–1.17), *p* < 0.001	15.0 (10.0–22.0)	8.0 (6.0–12.0)	9.0 (6.0–13.0)	Length of hospitalization (days), median (IQR)
1.28 (1.21–1.35), *p* < 0.001	2.0 (1.0–3.0)	1.0 (0.0–2.0)	1.0 (0.0–2.0)	SOFA score, median (IQR)
0.94 (0.92–0.95), *p* < 0.001	89.0 (85.0–94.0)	94.0 (89.0–97.0)	93.0 (88.0–97.0)	Oxygen saturation (%), mean (IQR)
Comorbidities				
4.15 (2.76–6.20), *p* < 0.001	47 (13.4)	58 (3.6)	105 (5.4)	Dementia, n (%)
1.58 (0.96–2.50), *p* = 0.061	24 (7.0)	73 (4.5)	97 (5.0)	COPD, n (%)
1.31 (0.65–2.43), *p* = 0.418	12 (3.5)	43 (2.7)	55 (2.8)	Stomach ulcer, n (%)
0.61 (0.21–1.42), *p* = 0.298	5 (1.4)	38 (2.4)	43 (2.2)	Liver diseases, n (%)
1.49 (1.10–1.99), *p* = 0.009	70 (20.1)	239 (14.8)	309 (15.8)	Well-controlled diabetes, n (%)
1.93 (1.04–3.42), *p* = 0.029	16 (4.6)	42 (2.6)	58 (3.0)	Poorly controlled diabetes, n (%)
1.87 (1.03–3.27), *p* = 0.032	17 (4.9)	43 (2.7)	60 (3.1)	Paresis, n (%)
0.53 (0.13–1.52), *p* = 0.304	3 (0.9)	26 (1.6)	29 (1.5)	Chronic renal failure, n (%)
1.7 (1.13–2.51), *p* = 0.009	36 (10.4)	103 (6.4)	139 (7.1)	Neoplasm, n (%)
1.08 (0.66–1.69), *p* = 0.76	23 (6.6)	99 (6.2)	122 (6.3)	Asthma, n (%)
1.91 (1.01–3.46), *p* = 0.037	15 (4.4)	37 (2.3)	52 (2.7)	Immunosuppressive treatment, n (%)
2.52 (0.94–6.20), *p* = 0.051	7 (2.0)	13 (0.8)	20 (1.0)	Interstitial lung diseases, n (%)
1.84 (1.28–2.61), *p* = 0.001	48 (13.8)	128 (8.0)	176 (9.0)	Atrial fibrillation, n (%)
1.68 (1.33–2.13), *p* < 0.001	208 (59.4)	748 (46.5)	956 (48.8)	Hypertension, n (%)
1.25 (0.65–2.26), *p* = 0.478	13 (4.6)	53 (3.7)	66 (3.8)	Alcohol abuse, n (%)
0.96 (0.71–1.28), *p* = 0.78	66 (21.4)	329 (22.2)	395 (22.0)	Nicotine addiction, n (%)
1.27 (0.34–8.25), *p* = 0.756	107 (30.4)	505 (31.3)	612 (31.1)	BMI > 30, n (%)
General symptoms				
0.77 (0.59–1.00), *p* = 0.045	238 (70.4)	1205 (75.6)	1443 (74.7)	Fever, n (%)
0.71 (0.55–0.91), *p* = 0.007	112 (34.1)	663 (42.1)	775 (40.8)	Myalgia, n (%)
0.62 (0.42–0.89), *p* = 0.013	34 (10.4)	249 (15.8)	283 (14.9)	Sore throat, n (%)
0.59 (0.39–0.88), *p* = 0.013	28 (8.6)	214 (13.6)	242 (12.8)	Running nose, n (%)
0.67 (0.51–0.88), *p* = 0.004	245 (73.8)	1286 (80.8)	1531 (79.6)	Cough, n (%)
1.38 (1.08–1.77), *p* = 0.012	228 (67.7)	962 (60.3)	1190 (61.6)	Dyspnoea, n (%)
0.72 (0.50–1.00), *p* = 0.054	45 (13.7)	286 (18.2)	331 (17.4)	Chest pain, n (%)
0.59 (0.20–1.38), *p* = 0.274	5 (1.5)	40 (2.5)	45 (2.4)	Haemoptysis, n (%)
0.62 (0.45–0.84), *p* = 0.003	55 (16.8)	386 (24.5)	441 (23.2)	Loss of taste, n (%)
0.53 (0.37–0.73), *p* < 0.001	47 (14.3)	379 (24.1)	426 (22.4)	Loss of smell, n (%)
0.92 (0.70–1.19), *p* = 0.536	91 (27.7)	463 (29.5)	554 (29.2)	Headache, n (%)
0.82 (0.59–1.13), *p* = 0.237	52 (15.8)	291 (18.5)	343 (18.0)	Nausea and vomiting, n (%)
0.81 (0.61–1.08), *p* = 0.165	68 (20.6)	381 (24.2)	449 (23.6)	Diarrhoea, n (%)
0.68 (0.42–1.04), *p* = 0.09	23 (7.0)	157 (10.0)	180 (9.5)	Abdominal pain, n (%)
1.08 (0.82–1.43), *p* = 0.584	253 (76.0)	1186 (74.5)	1439 (74.8)	Weakness, n (%)
Laboratory and radiological tests				
1.0 (1.00–1.00), *p* < 0.001	78.0 (46.0–184.5)	58.0 (30.0–150.0)	62.0 (33.0–156.5)	C-reactive protein, median (IQR)
1.75 (1.32–2.30), *p* < 0.001	85 (24.2)	246 (15.5)	331 (17.0)	EGFR < 60, n (%)
1.02 (1.01–1.04), *p* < 0.001	18.2 (13.6–24.9)	16.2 (11.9–21.6)	16.6 (12.2–22.1)	Lactate dehydrogenase (per 20), median (IQR)
1.06 (1.04–1.08), *p* < 0.001	7.0 (4.9–9.6)	5.5 (4.2–7.6)	5.7 (4.3–8.0)	Urea, median (IQR)
1.02 (1.00–1.07), *p* < 0.001	2.6 (1.7–4.9)	2.0 (1.3–3.4)	2.1 (1.3–3.6)	D-dimer (per 500), median (IQR)
1.03 (1.00–1.07), *p* = 0.106	0.1 (0.0–0.3)	0.1 (0.0–0.1)	0.1 (0.0–0.2)	Procalcitonin, median (IQR)
1.0 (1.00–1.00), *p* < 0.001	55.3 (26.5–111.1)	38.5 (16.3–78.5)	41.2 (17.6–84.0)	Interleukin-6, median (IQR)
1.03 (1.02–1.05), *p* < 0.001	1.0 (0.3–2.8)	0.3 (0.1–1.2)	0.4 (0.1–1.5)	NT-proBNP (per 500), median (IQR)
1.0 (0.86–1.14), *p* = 0.981	0.3 (0.0–0.8)	0.2 (0.0–0.6)	0.2 (0.0–0.6)	Proteinuria, median (IQR)
1.0 (0.95–1.02), *p* = 0.772	0.8 (0.6–1.1)	0.9 (0.7–1.4)	0.9 (0.6–1.3)	Lymphocytes, median (IQR)
1.07 (1.03–1.10), *p* < 0.001	5.5 (3.5–7.7)	4.7 (3.3–6.7)	4.8 (3.3–6.9)	Neutrophils, median (IQR)
1.01 (1.01–1.02), *p* < 0.001	40.0 (23.5–60.0)	30.0 (20.0–50.0)	30.0 (20.0–50.0)	High-resolution computed tomography (HRCT) lung injury (%), median (IQR)
Treatment				
4.72 (3.28–7.01), *p* < 0.001	320 (90.9)	1096 (67.9)	1416 (72.1)	Oxygen therapy, n (%)
3.1 (2.34–4.17), *p* < 0.001	285 (81.7)	944 (59.0)	1229 (63.0)	Steroid use, n (%)
2.14 (1.48–3.20), *p* < 0.001	311 (90.7)	1289 (81.9)	1600 (83.5)	Heparin use, n (%)
Complication during hospitalization				
2.54 (1.70–3.73), *p* < 0.001	42 (12.0)	82 (5.1)	124 (6.3)	Arrhythmia, n (%)
2.94 (1.59–5.31), *p* < 0.001	18 (5.1)	29 (1.8)	47 (2.4)	Cardiovascular (myocardial infarction, stroke), n (%)
2.33 (1.51–3.52), *p* < 0.001	35 (9.9)	73 (4.5)	108 (5.5)	Thromboembolism, n (%)

**Table 2:** Multivariate logistic regression model of factors related to developing infectious complications during hospitalization.

*p*-Value	OR CI	OR	Characteristics
*p* = 0.016	1.04–1.42	1.21	Age, mean (SD)
*p* < 0.001	1.10–1.15	1.12	Length of hospitalization, mean (SD)
*p* = 0.048	1.00–1.02	1.01	D-dimer, mean (SD)

ORs per increase in decade for age, day for length of hospitalization, or 500 units for D-dimer as the quantitative variable are reported, assuming linearity of the logit.

## Data Availability

The data that support the findings of this study will be available from the authors upon reasonable request, subject to applicable ethical and data protection regulations.
